# Congenital Anomalies in Children of Mothers Taking Antiepileptic Drugs with and without Periconceptional High Dose Folic Acid Use: A Population-Based Cohort Study

**DOI:** 10.1371/journal.pone.0131130

**Published:** 2015-07-06

**Authors:** Lu Ban, Kate M. Fleming, Pat Doyle, Liam Smeeth, Richard B. Hubbard, Linda Fiaschi, Laila J. Tata

**Affiliations:** 1 Division of Epidemiology & Public Health, University of Nottingham, Nottingham, United Kingdom; 2 Department of Non-communicable Disease Epidemiology, London School of Hygiene & Tropical Medicine, London, United Kingdom; Kuopio University Hospital, FINLAND

## Abstract

**Background:**

Antenatal antiepileptic drug (AED) use has been found to be associated with increased major congenital anomaly (CA) risks. However whether such AED-associated risks were different according to periconceptional high dose (5mg daily) folic acid supplementation is still unclear.

**Methods:**

We included 258,591 singleton live-born children of mothers aged 15-44 years in 1990-2013 from The Health Improvement Network, a large UK primary care database. We identified all major CAs according to the European Surveillance of Congenital Anomalies classification. Absolute risks and adjusted odds ratios (aOR) were calculated comparing children of mothers prescribed AEDs to those without such prescriptions, stratified by folic acid prescriptions around the time of conception (one month before conception to two months post-conception).

**Results:**

CA risk was 476/10,000 in children of mothers with first trimester AEDs compared with 269/10,000 in those without AEDs equating to an aOR of 1.82, 95% confidence interval 1.30-2.56. The highest system-specific risks were for heart anomalies (198/10,000 and 79/10,000 respectively, aOR 2.49,1.47-4.21). Sodium valproate and lamotrigine were both associated with increased risks of any CA (aOR 2.63,1.46-4.74 and aOR 2.01,1.12-3.59 respectively) and system-specific risks. Stratification by folic acid supplementation did not show marked reductions in AED-associated risks (e.g. for CAs overall aOR 1.75, 1.01-3.03 in the high dose folic acid group and 1.94, 95%CI 1.21-3.13 in the low dose or no folic acid group); however, the majority of mothers taking AEDs only initiated high dose folic acid from the second month of pregnancy.

**Conclusions:**

Children of mothers with AEDs in the first trimester of pregnancy have a 2-fold increased risk of major CA compared to those unexposed. We found no evidence that prescribed high dose folic acid supplementation reduced such AED-associated risks. Although statistical power was limited, prescribing of folic acid too late for it to be effective during the organogenic period or selective prescribing to those with more severe morbidity may explain these findings.

## Introduction

Epilepsy is a chronic neurological disorder affecting about 0.5% of women of childbearing age.[1] Women with epilepsy generally require ongoing antiepileptic drug (AED) treatment to remain seizure-free. This is particularly important when women become pregnant or are planning pregnancy since seizures in pregnancy pose unfavourable consequences not only to women themselves but also to the fetus.[2] However, increased risks of congenital anomalies (CAs), especially neural tube defects (NTDs), with antenatal exposure to AEDs (in particular valproate) have been reported across different study populations.[3–9] Whilst clinical guidelines from both the United Kingdom (UK)[10] and United States (US)[11] advise to avoid the use of valproate in pregnancy, the potential teratogenicity of other commonly prescribed AEDs such as carbamazepine has also been highlighted in recent studies.[6,12,13] Although newer-generation AEDs like lamotrigine and levetiracetam have not shown increased risks, study power to assess them has been limited.[14,15] This therefore poses a dilemma to both health care providers and women themselves with regards to drug treatment in pregnancy and a considerable proportion of women discontinue receiving their AED prescriptions when they become pregnant.[16]

Despite the lack of consistent evidence regarding the safety of individual AEDs, women treated with any AEDs are advised to take high dose (5mg) folic acid supplements daily before pregnancy throughout the first trimester to reduce the occurrence of NTDs and possibly other congenital anomalies such as heart anomalies and orofacial clefts.[17] Evidence for the effectiveness of such supplementation in women taking AEDs is scarce and previous observational studies have generally showed no protective effect.[13,18–21] These studies however investigated the effects of folic acid supplementation at any dose and were not limited to high dose alone. Furthermore, although reasonably sized, they used study populations that were recruited and provided informed consent during[18,20,21] or after[13,19] pregnancy and as such may not have been fully representative of the general population prescribed AEDs or folic acid.

Using data from UK primary care, we examined major CA risks in children of mothers prescribed any AEDs and individual drug types and assessed whether the risks were different according to whether mothers had received periconceptional high dose folic acid prescriptions.

## Methods

### Data source and study population

We used a pregnancy cohort study design which included all singleton live births for mothers aged 15–44 years between 1990 and 2013 from The Health Improvement Network (THIN), which is a nationally representative database of UK computerised primary care records containing validated medical diagnoses, events, symptoms and drug prescriptions and is widely used for pharmacoepidemiological research.[22] Anonymised children’s and mothers’ medical records were linked to provide prospectively recorded information before, during and after pregnancy. To ensure robust data on the timing of pre-conception period and gestational trimesters, we excluded children whose mothers had been registered for less than three months before conception or had no precise record of gestational week or an expected due date (166,578 were excluded; 39.2% of the original population). Since high dose folic acid supplementation is also recommended to mothers with existing diabetes because of an increased CA risk in their children and the concurrence of diabetes and epilepsy is extremely rare (e.g. 1.4% of mothers with epilepsy also had diabetes in our study population), we excluded children of mothers with a diagnosis of diabetes or prescriptions for anti-diabetic medication before or during pregnancy.

### Ethical approval

All data are anonymised, such that individual patients as well as the name and specific location of general practices cannot be identified by researchers. Ethical approval for this study was obtained from the Medical Research Ethics Committee, administered and approved by the National Health Service South East Research Ethics Committee (REC reference 04/MRE01/9)

### Outcome definitions

As described in detail previously,[23,24] we extracted all diagnostic recordings of major CAs (excluding genetic anomalies and anomalies attributed to known teratogens) from the children’s general practice (GP) records and classified these into system-specific groups based on the European Surveillance of Congenital Anomalies (EUROCAT) classification.[25] System-specific anomalies of heart, limbs and genital system (the three most common anomaly groups), the nervous system (anomalies that are most likely to be reduced following folic acid supplementation[26,27]) and other major anomalies were coded in this way. We had a median of four years of follow-up data after childbirth and we included CAs diagnosed in children up to age 20 years, where available.

### Exposure definitions

Exposure was defined as receipt of AEDs (British National Formulary (BNF) 4.8.1[28]) by mothers during pregnancy based on the presence/absence of a relevant drug prescription in mothers’ primary care electronic health records from four weeks before conception to childbirth. This was subsequently divided to AED prescriptions in the 1^st^ trimester of pregnancy (from four weeks before conception up to the end of 1^st^ trimester) or AEDs only in the 2^nd^ or 3^rd^ trimester. We also extracted prescription information for individual drugs, including carbamazepine, lamotrigine, sodium valproate, gabapentin, levetiracetam, phenytoin, pregabalin, clonazepam, clobazam, topiramate, phenobarbital, ethosuximide, vigabatrin, primidone, zonisamide, and lacosamide. We classified AEDs into two groups (using previous literature[29]): 1) older AEDs (those drugs approved before 1990) including carbamazepine, sodium valproate, phenytoin, clonazepam, clobazam, phenobarbital, ethosuximide and primidone and 2) newer AEDs (those approved after 1990) including lamotrigine, gabapentin, levetiracetam, pregabalin, topiramate, vigabatrin, zonisamide, and lacosamide. Furthermore, we examined the indication for AEDs by extracting recordings on epilepsy, serious mental illness, and other non-epileptic neurological conditions (i.e. migraine, neuralgia, neuropathic pain and essential tremor) before or during pregnancy.

### Folic acid prescription

From mothers’ records, we extracted all prescriptions for folic acid. Mothers were defined as having been prescribed periconceptional folic acid supplementation if they had one or more prescriptions for any folic acid in the month before conception (two weeks before the first day of the last menstrual period) or in the first eight weeks of pregnancy. In the UK 0.4mg and 5mg tablets are the most prescribed dose of folic acid. Although folic acid can be directly purchased from retailers, 5mg folic acid can only be obtained via a doctor’s prescription[28]. Based on the prescriptions, mothers were classified as having high dose folic acid if they had one or more folic acid prescriptions for at least 5mg daily, as having low dose folic acid if they had only folic acid prescriptions of less than 5mg daily, and no folic acid if they had no prescriptions of folic acid periconceptionally. Mothers with prescriptions of folic acid but without information on dosage were included in a separate category. All categories were mutually exclusive.

### Other co-variables

We extracted the year of the child’s birth (categorised as 1990–1995, 1996–2001, 2002–2007 and 2008–2013), maternal age at childbirth (categorised as age 15–24, 25–34 and 35–44 years), most recent maternal smoking status before delivery, most recent maternal body mass index (BMI) measurement before pregnancy (classified as normal, underweight, overweight and obese according to WHO classification[30]) and socioeconomic status as measured by quintiles of the Townsend Index of Deprivation.

### Statistical analysis

Absolute risks (per 10,000 live-born singletons) of any major CA and system-specific groups were calculated for children of mothers without AEDs in pregnancy, with AEDs in the first trimester and, as a separate control group, AEDs in the second or third trimester only. Logistic regression was used to estimate odds ratios (ORs) with 95% confidence intervals (95%CIs) for any CA and for heart, limbs, genital system and nervous system CAs, except where there were less than five exposed cases. The generalised estimating equation approach with exchangeable correlation structure was applied to take account of potential clustering between children born to the same woman in consecutive pregnancies. We also adjusted the ORs for maternal age, year of childbirth, maternal smoking, BMI and socioeconomic status. For children of mothers prescribed AEDs in the first trimester, we divided them into children of mothers with monotherapy and with polytherapy (more than one type of AEDs) and repeated the analysis. We then stratified the data according to whether mothers had been prescribed high dose folic acid or not, and re-calculated the absolute and relative risks of CAs in children of mothers prescribed AEDs (monotherapy or polytherapy) in the first trimester compared with children of mothers without AEDs in pregnancy in the two folic acid strata. We repeated the analyses for children of mothers prescribed carbamazepine, valproate, lamotrigine, older AEDs other than valproate and carbamazepine, or newer AEDs other than lamotrigine (the latter ones grouped because of low numbers).

To examine the prescribing pattern of folic acid around early pregnancy in UK primary care among mothers with first trimester AED prescriptions, we calculated the monthly prevalence of mothers with prescriptions of folic acid from three months before pregnancy to the end of the first trimester.

### Sensitivity analyses

We conducted three additional analyses to ensure the robustness of the study results. Firstly, to exclude the potential impact of the underlying disease, we excluded women prescribed AEDs in pregnancy for conditions other than epilepsy. Secondly, since only a small proportion of women prescribed high dose folic acid had it throughout the whole periconceptional period we restricted our study population to women prescribed high dose folic acid in the month before conception and throughout the first eight weeks of pregnancy. Thirdly, as a sensitivity analysis of individual AEDs, we restricted to children of mothers with monotherapy only.

All analyses were carried out using Stata SE 11.0 (Stata Corp., College Station, TX, US).

### Sample size calculation

Previous literature estimates that the prevalence of CA is 2.8%[24] and the prevalence of mothers prescribed AEDs in pregnancy is 0.5%.[16] Based on these numbers, we calculated that at least 76,953 children were needed to detect an OR of 2.0 for the association of CAs with antenatal AED exposure, with 80% power at a 5% significance level. The required sample size to achieve 80% power at a 5% significance level for system-specific anomalies was much larger (257,105 children were needed for heart anomalies based on our study population’s prevalence of 0.8%, 408,514 for limb anomalies based on its prevalence of 0.5%, 509,459 for genital anomalies based on its prevalence of 0.4% and 1,014,199 for nervous system anomalies based on its prevalence of 0.2%). Sample size calculations were performed using G*Power 3.1.7S.[31]

## Results

There were 258,591 singleton live-born children in the study and 1,438 whose mothers had prescriptions of AEDs in pregnancy. Of 1,259 children of mothers prescribed AEDs in the first trimester, 1,032 had monotherapy and 227 had polytherapy, and 450 were prescribed carbamazepine, 291 prescribed valproate, 155 prescribed other older AEDs, 389 prescribed lamotrigine and 217 prescribed other newer AEDs. Among mothers prescribed AEDs in pregnancy, the majority (81%) had epilepsy, 4% had serious mental illness, 16% had non-epileptic neurological conditions, 14% had more than one type of diagnosis and 11% had no diagnostic indication in their medical records. Proportionally more mothers were obese or overweight if they were prescribed AEDs in the first trimester than if not prescribed AEDs in pregnancy (Table 1). Mothers with AEDs in the first trimester were more likely to be smokers than mothers with AEDs only in later pregnancy or mothers without AEDs. Children of mothers with AED prescriptions were also more likely to be from socioeconomically deprived areas (Table 1).

**Table 1:** Maternal characteristics for children according to their mothers’ prescriptions of antiepileptic drugs in pregnancy (N = 258,591).

	No AEDs in pregnancy		AEDs in the 1^st^ trimester		AEDs only in the 2^nd^ or 3^rd^ trimester	
	n = 257,153		n = 1,259		n = 179	
	n	%	n	%	n	%
**Maternal Age, years**						
15–24	50,246	19.54	222	17.63	43	24.02
25–34	152,447	59.28	764	60.68	105	58.66
35–44	54,460	21.18	273	21.68	31	17.32
**Body Mass Index (kg/m** ^**2**^ **)**						
Normal (18.5–24.9)	116,794	58.07	510	50.00	82	59.85
Underweight (<18.5)	8,669	4.31	42	4.12	5	3.65
Overweight (25–29.9)	47,646	23.69	251	24.61	18	13.14
Obese (> = 30)	28,009	13.93	217	21.27	32	23.36
Missing*	56,035	[21.79]	239	[18.98]	42	[23.46]
**Maternal smoking**						
Non-smokers	164,544	81.10	771	74.93	107	79.85
Smokers	38,346	18.90	258	25.07	27	20.15
Missing*	54,263	[21.10]	230	[18.27]	45	[25.14]
**Townsend Deprivation Index**						
1 (least deprived)	60,602	25.28	235	20.40	30	17.34
2	47,865	19.96	195	16.93	19	10.98
3	51,148	21.33	231	20.05	31	17.92
4	46,386	19.35	275	23.87	51	29.48
5 (most deprived)	33,769	14.08	216	18.75	42	24.28
Missing*	17,383	[6.76]	107	[8.50]	6	[3.35]
**Indication for AED prescriptions**						
Epilepsy	—-		1,024	81.33	144	80.45
Serious mental illness	—-		54	4.29	8	4.47
Non-epileptic neurological conditions^a^	—-		202	16.04	25	13.97
Indication unknown	—-		139	11.04	26	14.53
**Folic acid prescriptions** ^**b**^						
None	217,355	84.52	502	39.87	125	69.83
Less than 5mg daily	35,529	13.82	104	8.26	24	13.41
At least 5mg daily	4,135	1.61	653	51.87	30	16.76
Dosage unknown	134	0.05	0	0.00	0	0.00

* Percentages in square brackets were calculated when including missing data;

^a^ migraine, neuralgia, neuropathic pain and essential tremor in the year before and or during pregnancy;

^b^ prescriptions of folic acid in two weeks before pregnancy or in the first eight weeks of pregnancy; AEDs = antiepileptic drugs

**Fig 1:**
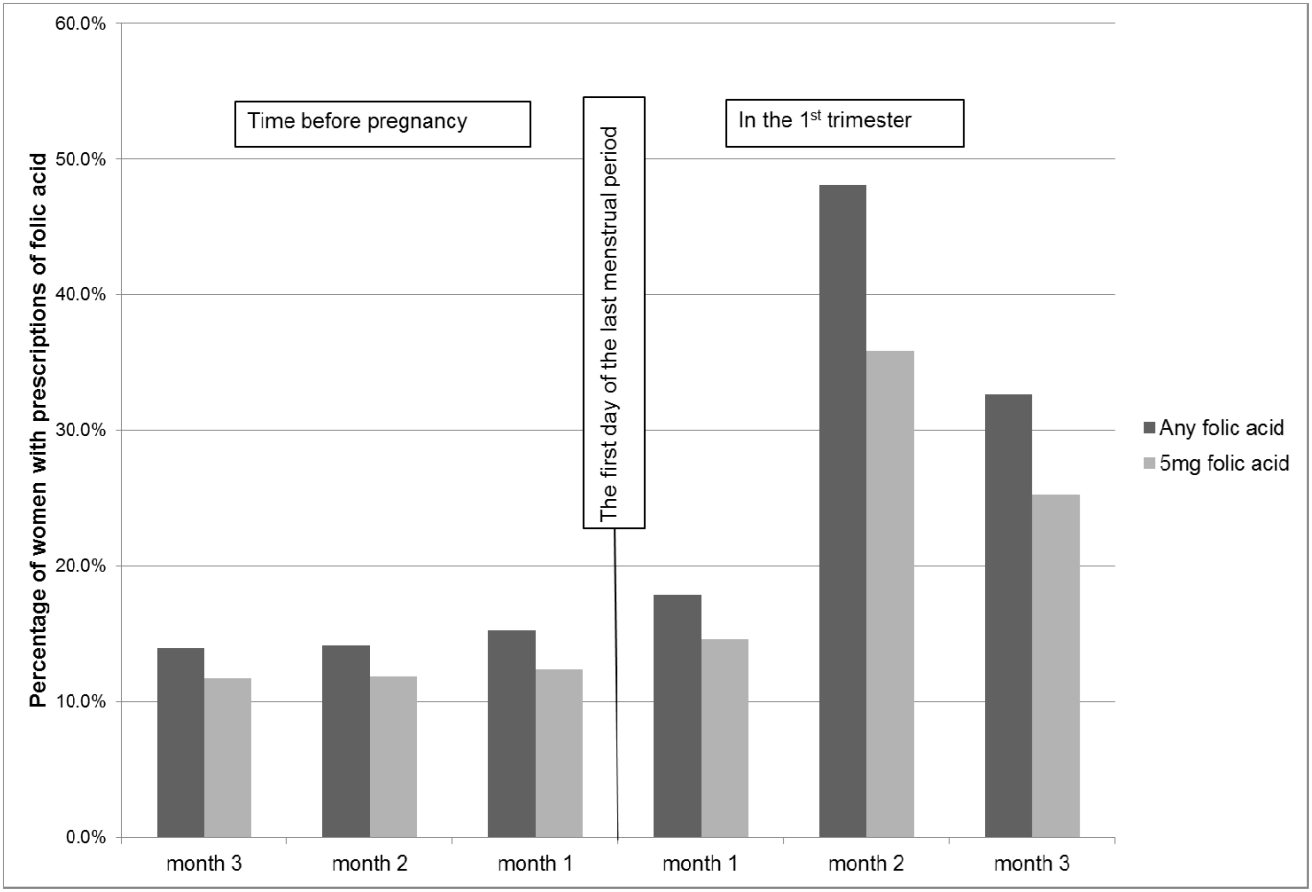
Percentages of women prescribed folic acid among those with first trimester antiepileptic drug prescriptions (N = 1,259). Monthly prevalence of any folic acid and 5mg folic acid prescribed in the three months before pregnancy and in the first trimester.

In mothers prescribed AEDs in the first trimester, 51.9% had high dose folic acid prescribed periconceptionally (Table 1) and the average quantity of each prescription was 30 tablets (interquartile range 28–56). There was a much lower proportion in those of mothers without AEDs (1.6%) or with AEDs only in later pregnancy (13.4%) (Table 1). When examining the monthly prevalence of folic acid prescriptions in those with AEDs in early pregnancy, less than 20% had a prescription for any folic before or in the first month of pregnancy; however, in the second month of pregnancy, the number increased considerably to nearly 50% (Fig 1). A similar pattern was observed for prescriptions of high dose folic acid (Fig 1).

The overall major CA risks were 269 per 10,000 in children of mothers without AEDs in pregnancy, 476 per 10,000 in children of mothers prescribed AEDs in the first trimester (Table 2) and 279 per 10,000 in those prescribed AEDs only in later pregnancy. Compared to children of mothers without AEDs, the ORs for the overall CA risk were 1.81 (95%CI 1.29–2.55) for children of mothers prescribed AEDs in the first trimester (Table 3) and 1.05 (95%CI 0.33–3.36) for children of mothers prescribed AEDs in later pregnancy only. The absolute system-specific CA risks in children exposed to AEDs in the first trimester were 198 per 10,000 for heart (OR 2.54, 95%CI 1.54–4.29 compared to unexposed), 79 for limb (OR 1.62, 95%CI 0.71–3.67), 87 for genital system (OR 2.24, 95%CI 1.02–4.89) and 32 for nervous system CAs (Tables 2 and 3). The adjusted ORs were very similar to the unadjusted for both overall CAs and system-specific groups (Table 3) and the results remained almost the same after excluding women prescribed AEDs in pregnancy for conditions other than epilepsy (S1 Table). The CA risks were higher for polytherapy than for monotherapy (Table 2). There was a 2.7-fold increased risk of the overall CA risk in children of mothers with AED polytherapy compared with children of mothers without AEDs in pregnancy (adjusted OR = 2.71, 95%CI 1.39–5.29) (Table 3).

**Table 2:** Absolute risks (per 10,000 children) of major congenital anomalies in children according to their mothers’ prescriptions of antiepileptic drugs in pregnancy*.

	No AEDs in pregnancy		AEDs in the 1^st^ trimester					
			Overall		Monotherapy		Polytherapy^a^	
	n = 257,153		n = 1,259		n = 1,032		n = 227	
	n	*n/10*,*000*	n	*n/10*,*000*	n	*n/10*,*000*	n	*n/10*,*000*
**Any major anomaly**	6,922	*269*	60	*476*	44	*426*	16	*705*
Heart	2,041	*79*	25	*198*	20	*194*	5	*220*
Limb	1,263	*49*	10	*79*	7	*68*	3	*132*
Genital system	1,023	*40*	11	*87*	7	*68*	4	*176*
Nervous system	367	*14*	4	*32*	3	*29*	1	*44*
Other anomalies^b^	2,872	*112*	17	*135*	14	*136*	4	*176*

*Results for 179 children born to women with AEDs only in the 2^nd^ or 3^rd^ trimester, of which 5 had major congenital anomalies, are presented in the text only;

^a^ more than one type of antiepileptic drug was prescribed;

^b^ All other major congenital anomalies not classified as heart, limb, genital system, or nervous system;

AEDs = antiepileptic drugs

**Table 3:** Odds ratios for the association of major congenital anomalies with antiepileptic drugs in the 1^st^ trimester of pregnancy and risk stratification according to whether high dose (5mg daily) folic acid was prescribed*.

	AEDs in the 1^st^ trimester							
	Overall				Monotherapy		Polytherapy^a^	
	cOR	95%CI	aOR	95%CI	aOR	95%CI	aOR	95%CI
**Overall population**		**n exposed = 1,259**			**n exposed = 1,032**		**n exposed = 227**	
Any major anomaly	1.81	1.29–2.55	1.82	1.30–2.56	1.62	1.10–2.42	2.71	1.39–5.29
Heart	2.54	1.51–4.29	2.49	1.47–4.21	2.44	1.36–4.37	2.70	0.84–8.70
Limb	1.62	0.71–3.67	1.66	0.73–3.75	1.42	0.54–3.75	-	
Genital system	2.24	1.02–4.89	2.28	1.04–4.99	1.78	0.67–4.72	-	
Nervous system	-		-		-		-	
**Prescriptions of folic acid At least 5mg daily**		**n exposed = 653**			**n exposed = 521**		**n exposed = 132**	
Any major anomaly	1.63	0.95–2.80	1.75	1.01–3.03	1.56	0.83–2.92	2.54	0.99–6.54
Heart	2.71	1.19–6.21	3.25	1.41–7.52	3.07	1.25–7.55	-	
Limb	-		-		-		-	
Genital system	2.12	0.63–7.18	2.18	0.61–7.84	-		-	
Nervous system	-		-		-		-	
**None/less than 5mg daily**		**n exposed = 606**			**n exposed = 511**		**n exposed = 95**	
Any major anomaly	1.96	1.22–3.15	1.94	1.21–3.13	1.71	0.98–2.96	3.22	1.23–8.44
Heart	2.31	1.05–5.08	2.22	1.10–6.92	2.16	0.91–5.16	-	
Limb	2.72	1.09–6.83	2.76	1.12–7.01	2.05	0.64–6.55	-	
Genital system	2.11	0.66–6.69	2.15	0.68–6.82	-		-	
Nervous system	-		-		-		-	

* 134 children whose mothers’ were prescribed folic acid had no information on dosage. Empty cells indicate there were fewer than five exposed cases, for which statistical analyses were not performed;

^a^ more than one type of antiepileptic drug was prescribed;

cOR = crude odds ratio; aOR = odds ratio adjusted for maternal age, year of childbirth, maternal body mass index, smoking and socioeconomic status;

AEDs = antiepileptic drugs; 95%CI = 95% confidence interval

When stratifying the analysis by children of mothers with and without prescriptions of high dose folic acid around early pregnancy, the adjusted ORs were similar (Table 3). When restricting to children of mothers with high dose folic acid throughout the whole periconceptional period, we found that only 66 women with AEDs in the first trimester had high dose folic acid prescribed throughout the whole periconceptional period and less than five had a major congenital anomaly of which none was nervous system anomaly (adjusted OR = 1.52, 95%CI 0.16–14.16 compared to children of women without AEDs for the overall CA risk).

When assessing the effects for individual AEDs (Table 4), the absolute risks of overall CAs were generally highest in children of mothers prescribed valproate (687 per 10,000) and other old AEDs combined (710 per 10,000), followed by the risks in those of mothers prescribed newer drugs (514 per 10,000 for lamotrigine and 369 per 10,000 for other newer drugs combined). The pattern was similar for system-specific anomalies (Table 4). Compared with children of mothers without AEDs, the adjusted ORs of overall CAs were statistically significant for valproate (2.63, 95%CI 1.46–4.73), lamotrigine (2.01, 1.12–3.59) and other older AEDs (2.67, 1.18–6.04) but not for carbamazepine (1.58, 0.86–2.89) and other newer AEDs (1.44, 0.57–3.65) (S2 Table). The results for the monotherapy of individual AEDs were similar to the main analysis (S3 Table).

**Table 4:** Absolute risks (per 10,000 children) of major congenital anomalies in children according to type of antiepileptic drug in the 1^st^ trimester of pregnancy.

	Individual types of AEDs in the 1^st^ trimester of pregnancy									
	Carbamazepine		Sodium valproate		Other older AEDs^a^ combined		Lamotrigine		Other newer AEDs^b^ combined	
	n = 450		n = 291		n = 155		n = 389		n = 217	
	n	*n/10*,*000*	n	*n/10*,*000*	n	*n/10*,*000*	n	*n/10*,*000*	n	*n/10*,*000*
**Any major anomaly**	19	*422*	20	*687*	11	*710*	20	*514*	8	*369*
Heart	8	*178*	6	*206*	5	*323*	9	*231*	2	*92*
Limb	2	*44*	5	*172*	1	*65*	4	*103*	0	-
Genital system	5	*111*	4	*137*	3	*194*	2	*51*	3	*138*
Nervous system	2	*44*	3	*103*	0	-	1	*26*	0	-
Other anomalies^c^	5	*111*	4	*137*	4	*258*	5	*129*	4	*184*

^a^ phenytoin, clonazepam, clobazam, phenobarbital, ethosuximide, or primidone;

^b^ gabapentin, levetiracetam, pregabalin, topiramate, vigabatrin, zonisamide, or lacosamide;

^c^ All other major congenital anomalies not classified as heart, limb, genital system, or nervous system;

AEDs = antiepileptic drugs

After stratifying the analysis by folic acid prescriptions (S2 Table), the lamotrigine-associated CA risk decreased in the group with high dose folic acid (adjusted OR = 1.60, 95%CI 0.66–3.93), but remained statistically significant in the group with no or low dose folic acid (2.89, 1.29–6.46). However, the confidence intervals of the two ORs overlapped.

## Discussion

### Principal findings

Compared with children of mothers without AEDs in pregnancy, the major CA risk was 2-fold higher in children of mothers prescribed AEDs in early pregnancy but not in later pregnancy. Such risks were especially evident in children of mothers prescribed polytherapy and valproate, but children of mothers prescribed lamotrigine also showed increased risks for overall and heart anomalies. Only about half of women prescribed AEDs in the first trimester were also prescribed high dose folic acid around early pregnancy with mostly initiated in the second month of pregnancy only. We found no evidence that periconceptional high dose folic acid as prescribed in this population reduced the CA risk associated with AED prescriptions, although this may reflect late prescribing or selective prescribing to women with severe conditions.

### Strengths and limitations

Our study is the first UK study to examine the risk of CAs in children born to women with AEDs in pregnancy compared to children born to women without AEDs in the general population which is not readily available in studies using data from epilepsy registries.[8,14,18,32] Our data on exposure, outcome and co-variables were gathered from UK GP recorded prospectively in the course of routine clinical care, thus minimising recall and reporting bias. Although our overall sample size is comparable to previous population-based studies of AED teratogenicity,[18–20] the numbers for specific anomaly groups with individual AEDs are inevitably low, especially after we stratified the analysis by periconceptional folic acid prescriptions. We only presented the ORs where there were at least five exposed cases therefore did not provide effect measures for all specific anomaly groups.

Regarding the completeness of recording for drug prescriptions in THIN, although most drug prescriptions are issued in primary care it is possible that women without well-controlled epilepsy may have been referred to epilepsy specialist clinics and subsequently received prescriptions there. However, all pregnant women in the UK must be registered with a GP to benefit from free antenatal care and prescriptions, and even if AED treatment is initiated by a neurologist, the prescribing would normally be carried out via the GP who is responsible for long-term follow-up and clinical management. Similarly, since 5mg tablets can only be issued with a prescription from a doctor,[28] it is unlikely that women with high dose (5mg) folic acid were not captured in THIN. As women on AEDs are clinically recommended high dose folic acid for the prevention of NTDs in pregnancy, we therefore believe that our assessment of this within THIN should be representative of practice and thus risks in the UK.

It must be recognised that women receiving prescriptions may not have actually taken the medication during the organogenetic period, which could bias our estimates to the null. Previous research for example has reported that the non-adherence to medication in patients with epilepsy is about 30–50%.[33] Studies large enough to assess congenital anomaly risks however are generally limited in their ability to obtain information on actual medication compliance. Therefore the true effect of AED exposure on the major congenital anomaly risk could be higher than most current estimates. Regarding folic acid use, one limitation of our study was that despite guidelines suggested that women with AEDs should take high dose folic acid before getting pregnant, only a small proportion of them had high dose folic acid throughout the whole periconceptional period. A previous study using data from the UK Epilepsy and Pregnancy Register[18] found that 45% of women with AEDs also reported taking folic acid supplements (at any dose) before conception. The women included in this registry study[18] were either self-referred or referred by their doctors or midwives when pregnant and the information on the folic acid supplementation was subsequently collected retrospectively depending on women’s self-disclosure. Therefore some women might only initiate folic acid supplementation immediately after they became pregnant. Although our study found a slightly lower prevalence of women with folic acid prescriptions before conception than the registry study, the proportions are very similar when we include the prescriptions of folic acid in the first eight weeks of pregnancy, which reassuringly indicates that prescribing generally reflects use. After we restricted to women with high dose folic acid prescribed for the whole periconceptional period, we found the estimate for the overall CA risk associated with the antenatal AED exposure was similar to the main analysis.

Regarding the completeness of capture of anomalies, we had a median of four years of follow-up data after childbirth and we included CAs diagnosed in children up to age 20 years, where available. We thus expect to have captured CAs for live births as completely if not more completely than registry data.[24] As stillborn children are not registered with a GP they are excluded from this study as in most previous studies. Stillbirth occurs in approximately 0.6% of births in developed countries,[34] of whom 8–14% have congenital anomalies[35,36] and the effect of excluding them on our estimates should be minimal. We were also unable to ascertain CAs among pregnancies ending in spontaneous or induced abortions. It is likely that a considerable proportion of pregnancies where the fetus has a severe CA would end in spontaneous abortion. There are no accurate data sources to ascertain congenital anomalies in these cases, as spontaneous abortion is likely to occur in early pregnancy when many women may not know they are pregnant, and autopsy information on later losses is rarely ascertained. This is similar for induced abortion (or medical/surgical termination) as most happen in early pregnancy. Based on estimates from EUROCAT,[37] the prevalence of termination of pregnancy for fetal anomalies due to NTDs is about 8 per 10,000 births in the UK. We therefore could miss at least one third of NTDs by only including live births in our study population. However, UK registry ascertainment of CAs among medically terminated pregnancies varies regionally[38] and national abortion statistics estimate only 1% are due to CA.[39] In addition, we do not have sufficient data on whether or not a woman would have a prior pregnancy affected with a NTD. It is possible that such women would be more likely to receive high dose folic acid for a subsequent pregnancy than women without a history. However, a recent systematic review showed that the risk of recurrent NTD was decreased statistically significantly after women taking a high dose of folic acid (4mg/day).[40] We believe that our results related to NTDs in this study would not be significantly affected by whether or not to control for the information of a prior history of NTD.

### Interpretation

Similar to previous studies, our study confirmed the increased risk of CAs, such as heart and genital anomalies, in children of mothers exposed to AED polytherapy and valproate in the first trimester of pregnancy compared with children of mothers who were unexposed.[19,32,41–46] A previous study from the UK Epilepsy and Pregnancy Register, for example, found higher risks of CAs in women with polytherapy (6.0%) and with valproate monotherapy (6.2%) than in women with other monotherapy and with epilepsy but without AEDs (3.5%).[32] In line with our results, the authors also found that women with carbamazepine had the lowest CA risk compared to other drugs.[32]

Although it has been proposed that AEDs may disturb folate metabolism through antagonizing enzymes in the folate methylation cycle, impairing folate absorption, or increasing folate degradation which all could in turn cause neural crest cell disruption,[47] the exact teratogenic mechanism of AEDs is still unclear. Previous research has suggested that patients with epilepsy treated with AEDs, such as valproate and carbamazepine, have lower folate levels than untreated patients or healthy controls.[48] Only five studies[13,18–21] (two conducted using the same study population and three from various registers) thus far have examined the effects of folic acid supplementation before or in early pregnancy on the AED-associated CA risks. The neural tube closes at six weeks of pregnancy (four weeks after conception) after which there would probably be little benefit of folic acid in preventing NTDs. A recent study examining approximately 1,900 pregnancies with folic acid supplementation initiated before conception[18] found no evidence that folic acid supplementation was protective in terms of the overall CA risk or the risk of NTDs in children of mothers exposed to AEDs. Similar results were also reported in another study using Australian Pregnancy Registry data.[20]

In addition, Hernández-Díaz and colleagues from the US conducted three studies to examine the safety of AEDs and individual drugs in pregnancy.[7,13,19] Using data from the North American AED Pregnancy Registry, the authors found higher risks of CAs in children of mothers with older AEDs such as valproate and phenobarbital than children of mothers with newer AEDs such as lamotrigine and levetiracetam.[7] In another two case-control studies[13,19] of mothers of children with congenital anomalies identified through maternity hospitals or tertiary care hospitals after pregnancy, the authors found that use of folic acid antagonists (including AEDs) in early pregnancy was associated with increased risks of heart defects, oral clefts and urinary tract defects compared to women without AEDs and, in line with our findings, folic acid supplementation in pregnancy did not modify these increased risks.[19] The authors (in a separate study) also reported a trend of decreasing risk of NTDs in the folic acid supplemented group but this finding was not statistically significant.[13] The data on drug exposures in the case-control studies were collected through self-reported questionnaires within six months after delivery, thus it is likely subject to recall bias and the exact timing and dosage of folic acid supplementation were not clear.

To our best knowledge, the only study thus far presenting detailed information on both timing and dosage of folic acid supplementation is a recent study using registry data from Netherlands.[21] This study identified women who used at least 0.4mg folic acid continuously from four weeks before conception until eight weeks in pregnancy.[21] Although this study found that intake of folic acid around early pregnancy had a protective effect in terms of reducing the overall risk of spina bifida in the AED-unexposed group, no such effect was found in pregnant women exposed to valproate.[21] This study however did not examine the effect of high dose folic acid supplementation explicitly thus cannot exclude any potential protective effect at the higher dose level.

It is also possible that women who were prescribed a higher dose of folic acid might have more severe underlying illness and/or were on higher dose AEDs for a substantially long period of time, which could obfuscate any true protective effects of high dose folic acid supplementation if it did exist. Such a limitation however is present in all observational studies.

### Conclusion

Children born to women with AEDs prescribed in the first trimester of pregnancy (in particular AED polytherapy and valproate) have increased risks of major CAs compared to the general population. Although folic acid may reduce the baseline risk of NTDs and other CAs in the general population, we found that the additional risk associated with the antenatal AED exposure is similar between children of mothers with and without periconceptional high dose folic acid as prescribed in this population. Despite guidelines indicating that all women on AEDs should receive high dose folic acid preconceptionally, this may be due to late folic acid prescribing or selective prescribing to women with more severe morbidity. Further research is needed to investigate the optimal dose and timing of high dose folic acid supplementation ideally in well-designed clinical trials. Meanwhile, since there are no harmful effects of low dose folic acid supplementation, women of childbearing age with AEDs should still be recommended to take folic acid supplementation as in the general population.

## Details of Ethical Approval

All data are anonymised, such that individual patients as well as the name and specific location of general practices cannot be identified by researchers. Ethical approval for this study was obtained from the Medical Research Ethics Committee, administered and approved by the National Health Service South East Research Ethics Committee (REC reference 04/MRE01/9).

## Supporting Information

S1 Table Sensitivity analysis of the association of major congenital anomalies with antiepileptic drugs in the 1^st^ trimester of pregnancy after excluding women prescribed antiepileptic drugs for conditions other than epilepsy (N = 258,321). (DOC)

S2 Table Adjusted odds ratios for the association of major congenital anomalies with individual antiepileptic drug types in the 1^st^ trimester of pregnancy and risk stratification according to whether high dose (at least 5mg daily) folic acid was prescribed*. (DOC)

S3 Table Sensitivity analysis of individual antiepileptic drug types prescribed as monotherapy only: Adjusted odds ratios for the association of major congenital anomalies with antiepileptic drugs in the 1^st^ trimester of pregnancy*. (DOC)
