# Supplementary material for: Congenital Anomalies in Children of Mothers Taking Antiepileptic Drugs with and without Periconceptional High Dose Folic Acid Use: A Population-Based Cohort Study
Source: PLoS One. 2015 Jul 6;10(7):e0131130. doi: 10.1371/journal.pone.0131130 (PMC4492893; doi:10.1371/journal.pone.0131130)
Supplement: S1 Table — (DOC) [file pone.0131130.s001.doc]

et al. Valproic acid monotherapy in pregnancy and major congenital malformations. New England Journal of Medicine. 2010;362: 2185–2193. doi:10.1056/NEJMoa0907328

6. Tomson T, Battino D, Bonizzoni E, Craig J, Lindhout D, Sabers A, et al. Dose-dependent risk of malformations with antiepileptic drugs: an analysis of data from the EURAP epilepsy and pregnancy registry. The Lancet Neurology. 2011;10: 609–617. doi:10.1016/S1474-4422(11)70107-7

7. Hernández-Díaz S, Smith CR, Shen A, Mittendorf R, Hauser WA, Yerby M, et al. Comparative safety of antiepileptic drugs during pregnancy. Neurology. 2012;78: 1692–1699. doi:10.1212/WNL.0b013e3182574f39

8. Mawhinney E, Campbell J, Craig J, Russell A, Smithson W, Parsons L, et al. Valproate and the risk for congenital malformations: Is formulation and dosage regime important? Seizure. 2012;21: 215–218. doi:10.1016/j.seizure.2012.01.005

9. Vajda FJ, O’Brien TJ, Graham JE, Lander CM, Eadie MJ. Dose dependence of fetal malformations associated with valproate. Neurology. 2013;81: 999–1003. doi:10.1212/WNL.0b013e3182a43e81

10. NICE. CG137 Epilepsy: full guideline. In: NICE [Internet]. [cited 17 May 2013]. Available: http://www.nice.org.uk/

11. Harden CL, Meador KJ, Pennell PB, Hauser WA, Gronseth GS, French JA, et al. Practice Parameter update: Management issues for women with epilepsy—Focus on pregnancy (an evidence-based review): Teratogenesis and perinatal outcomes Report of the Quality Standards Subcommittee and Therapeutics and Technology Assessment Subcommittee of the American Academy of Neurology and American Epilepsy Society. Neurology. 2009;73: 133–141. doi:10.1212/WNL.0b013e3181a6b312

12. Jentink J, Dolk H, Loane MA, Morris JK, Wellesley D, Garne E, et al. Intrauterine exposure to carbamazepine and specific congenital malformations: systematic review and case-control study. BMJ. 2010;341: c6581–c6581. doi:10.1136/bmj.c6581

13. Hernández-Díaz S, Werler MM, Walker AM, Mitchell AA. Neural tube defects in relation to use of folic acid antagonists during pregnancy. Am J Epidemiol. 2001;153: 961–968. doi:10.1093/aje/153.10.961

14. Mawhinney E, Craig J, Morrow J, Russell A, Smithson WH, Parsons L, et al. Levetiracetam in pregnancy: results from the UK and Ireland epilepsy and pregnancy registers. Neurology. 2013;80: 400–405. doi:10.1212/WNL.0b013e31827f0874

15. Mølgaard-Nielsen D HA. Newer-generation antiepileptic drugs and the risk of major birth defects. JAMA. 2011;305: 1996–2002. doi:10.1001/jama.2011.624

16. Man S-L, Petersen I, Thompson M, Nazareth I. Antiepileptic drugs during pregnancy in primary care: a UK population based study. PLoS ONE. 2012;7: e52339. doi:10.1371/journal.pone.0052339

17. NICE. CG62 Antenatal care: NICE guideline [Internet]. [cited 14 Jun 2011]. Available: http://guidance.nice.org.uk/CG62/NICEGuidance/pdf/English

18. Morrow JI, Hunt SJ, Russell AJ, Smithson WH, Parsons L, Robertson I, et al. Folic acid use and major congenital malformations in offspring of women with epilepsy: a prospective study from the UK Epilepsy and Pregnancy Register. J Neurol Neurosurg Psychiatry. 2009;80: 506–511. doi:10.1136/jnnp.2008.156109

19. Hernández-Díaz S, Werler MM, Walker AM, Mitchell AA. Folic acid antagonists during pregnancy and the risk of birth defects. New England Journal of Medicine. 2000;343: 1608–1614. doi:10.1056/NEJM200011303432204

20. Vajda FJE, Hitchcock A, Graham J, Solinas C, O’Brien TJ, Lander CM, et al. Foetal malformations and seizure control: 52 months data of the Australian Pregnancy Registry. European Journal of Neurology. 2006;13: 645–654. doi:10.1111/j.1468-1331.2006.01359.x

21. Jentink J, Bakker MK, Nijenhuis CM, Wilffert B, de Jong-van den Berg LTW. Does folic acid use decrease the risk for spina bifida after in utero exposure to valproic acid? Pharmacoepidemiology and Drug Safety. 2010;19: 803–807. doi:10.1002/pds.1975

22. Lewis JD, Schinnar R, Bilker WB, Wang X, Strom BL. Validation studies of the health improvement network (THIN) database for pharmacoepidemiology research. Pharmacoepidem Drug Safe. 2007;16: 393–401. doi:10.1002/pds.1335

23. Ban L, West J, Abdul Sultan A, Dhalwani N, Ludvigsson J, Tata L. Limited risks of major congenital anomalies in children of mothers with coeliac disease: a population-based cohort study. BJOG: Int J Obstet Gy. 2014; n/a–n/a. doi:10.1111/1471-0528.13102

24. Sokal R, Fleming KM, Tata LJ. Potential of general practice data for congenital anomaly research: comparison with registry data in the United Kingdom. Birth Defects Research Part A: Clinical and Molecular Teratology. 2013;97: 546–553. doi:10.1002/bdra.23150

25. EUROCAT. Coding of EUROCAT subgroups of congenital anomalies [Internet]. 2012. Available: http://www.eurocat-network.eu/content/EUROCAT-Guide-1.3-Chapter-3.3-Jan13.pdf

26. Prevention of neural tube defects: results of the Medical Research Council Vitamin Study. MRC Vitamin Study Research Group. Lancet. 1991;338: 131–137.

27. Czeizel AE, Dudás I. Prevention of the First Occurrence of Neural-Tube Defects by Periconceptional Vitamin Supplementation. New England Journal of Medicine. 1992;327: 1832–1835. doi:10.1056/NEJM199212243272602

28. Joint Formulary Committee. British National Formulary (BNF) 63. 63rd Revised edition. Pharmaceutical Press; 2012.

29. Ali M. Investigating the use of medicines in management of children and young people with epilepsy using data from primary care in the UK [Internet]. PhD, University of Nottingham. 2012. Available: http://etheses.nottingham.ac.uk/2898/

30. World Health Organization. BMI classification [Internet]. [cited 9 Jan 2014]. Available: http://apps.who.int/bmi/index.jsp?introPage=intro_3.html

31. Prajapati B, Dunne M, Armstrong R. Sample size estimation and statistical power analyses. Optometry Today. July 16. Available: http://www.optometry.co.uk/clinical/details?aid=634. Accessed 10 May 2012.

32. Morrow J, Russell A, Guthrie E, Parsons L, Robertson I, Waddell R, et al. Malformation risks of antiepileptic drugs in pregnancy: a prospective study from the UK epilepsy and pregnancy register. J Neurol Neurosurg Psychiatry. 2006;77: 193–198. doi:10.1136/jnnp.2005.074203

33. Leppik IE. How to get patients with epilepsy to take their medication. The problem of noncompliance. Postgrad Med. 1990;88: 253–256.

34. Say L, Donner A, Gülmezoglu AM, Taljaard M, Piaggio G. The prevalence of stillbirths: a systematic review. Reproductive Health. 2006;3: 1. doi:10.1186/1742-4755-3-1

35. The Stillbirth Collaborative Research Network. Causes of death among stillbirths. JAMA. 2011;306: 2459–2468. doi:10.1001/jama.2011.1823

36. Confidential Enquiry into Maternal and Child Health (CEMACH). Perinatal Mortality 2007: United Kingdom [Internet]. CEMACH: London 2009; Available: http://www.hqip.org.uk/assets/NCAPOP-Library/CMACE-Reports/37.-June-2009-Perinatal-Mortality-2007.pdf

37. European Surveillance of Congenital Anomalies. EUROCAT prevalence tables [Internet]. [cited 21 Apr 2015]. Available: http://www.eurocat-network.eu/accessprevalencedata/prevalencetables

38. BINOCAR. Congenital Anomaly Statistics 2010 England and Wales [Internet]. 2012. Available: http://www.binocar.org/content/Annual%20report%202010%20FINAL%2031_07_12%20v2.pdf

39. Department of Health. Abortion statistics, England and Wales, 2010 [Internet]. 24 May 2011 [cited 21 Jun 2012]. Available: http://www.dh.gov.uk/en/Publicationsandstatistics/Publications/PublicationsStatistics/DH_126769

40. Grosse SD, Collins JS. Folic acid supplementation and neural tube defect recurrence prevention. Birth Defects Research Part A: Clinical and Molecular Teratology. 2007;79: 737–742. doi:10.1002/bdra.20394

41. Thomas SV, Ajaykumar B, Sindhu K, Francis E, Namboodiri N, Sivasankaran S, et al. Cardiac malformations are increased in infants of mothers with epilepsy. Pediatr Cardiol. 2008;29: 604–608. doi:10.1007/s00246-007-9161-4

42. Kaaja E, Kaaja R, Hiilesmaa V. Major malformations in offspring of women with epilepsy. Neurology. 2003;60: 575–579.

43. Holmes LB, Harvey EA, Coull BA, Huntington KB, Khoshbin S, Hayes AM, et al. The teratogenicity of anticonvulsant drugs. N Engl J Med. 2001;344: 1132–8.

44. Queisser-Luft A, Eggers I, Stolz G, Kieninger-Baum D, Schlaefer K. Serial examination of 20,248 newborn fetuses and infants: correlations between drug exposure and major malformations. Am J Med Genet. 1996;63: 268–76.

45. Kulaga S, Sheehy O, Zargarzadeh AH, Moussally K, Bérard A. Antiepileptic drug use during pregnancy: Perinatal outcomes. Seizure. 2011;20: 667–672. doi:10.1016/j.seizure.2011.06.012

46. Cassina M, Dilaghi A, Di Gianantonio E, Cesari E, De Santis M, Mannaioni G, et al. Pregnancy outcome in women exposed to antiepileptic drugs: Teratogenic role of maternal epilepsy and its pharmacologic treatment. Reproductive Toxicology. 2013;39: 50–57. doi:10.1016/j.reprotox.2013.04.002

47. Gelder MMHJ van, Rooij IALM van, Miller RK, Zielhuis GA, Berg LTW de J den, Roeleveld N. Teratogenic mechanisms of medical drugs. Hum Reprod Update. 2010;16: 378–394. doi:10.1093/humupd/dmp052

48. Linnebank M, Moskau S, Semmler A, Widman G, Stoffel-Wagner B, Weller M, et al. Antiepileptic drugs interact with folate and vitamin B12 serum levels. Annals of Neurology. 2011;69: 352–359. doi:10.1002/ana.22229

)

|  | |  | |  | |  | |
| --- | --- | --- | --- | --- | --- | --- | --- |
|  | |  | |  | |
|  |  |  |  |  |  |
|  | |  |  |  |  |  |  |
|  | |  |  |  |  |  |  |
|  | |  |  |  |  |  |  |
|  | |  |  |  |  |  |  |
|  | |  |  |  |  |  |  |
|  | |  |  |  |  |  |  |
|  | |  |  |  |  |  |  |
|  | |  |  |  |  |  |  |
|  | |  |  |  |  |  |  |
|  | |  |  |  |  |  |  |
|  | |  |  |  |  |  |  |
|  | |  |  |  |  |  |  |
|  | |  |  |  |  |  |  |
|  | |  |  |  |  |  |  |
|  | | |  |  |  |  |  |
|  |  | |  |  |  |  |  |
|  |  | |  |  |  |  |  |
|  |  | |  |  |  |  |  |
|  |  | |  |  |  |  |  |
|  |  | |  |  |  |  |  |
|  |  | |  |  |  |  |  |
|  |  | |  |  |  |  |  |
|  |  | |  |  |  |  |  |
|  |  | |  |  |  |  |  |
|  |  | |  |  |  |  |  |
|  |  | |  |  |  |  |  |
|  |  | |  |  |  |  |  |
|  |  | |  |  |  |  |  |
|  |  | |  |  |  |  |  |
|  |  | |  |  |  |  |  |
|  |  | |  |  |  |  |  |

|  |  | |  | | | | | |
| --- | --- | --- | --- | --- | --- | --- | --- | --- |
|  | |  | |  | |
|  | |  | |  | |  | |
|  |  |  |  |  |  |  |  |
|  |  |  |  |  |  |  |  |  |
|  |  |  |  |  |  |  |  |  |
|  |  |  |  |  |  |  |  |  |
|  |  |  |  |  |  |  |  |  |
|  |  |  |  |  |  |  |  |  |
|  |  |  |  |  |  |  |  |  |

|  |  | | | | | | | | | |
| --- | --- | --- | --- | --- | --- | --- | --- | --- | --- | --- |
|  | | | |  |  | |  |  | |
|  |  |  |  |  |  |  |  |  |  |
|  |  | | | |  |  | |  |  | |
|  |  |  |  |  |  |  |  |  |  |  |
|  |  |  |  |  |  |  |  |  |  |  |
|  |  |  |  |  |  |  |  |  |  |  |
|  |  |  |  |  |  |  |  |  |  |  |
|  |  |  |  |  |  |  |  |  |  |  |
|  |  |  |  |  |  |  |  |  |  |  |
|  | |  | | |  |  | |  |  | |
|  |  |  |  |  |  |  |  |  |  |  |
|  |  |  |  |  |  |  |  |  |  |  |
|  |  |  |  |  |  |  |  |  |  |  |
|  |  |  |  |  |  |  |  |  |  |  |
|  |  |  |  |  |  |  |  |  |  |  |
|  | |  | | |  |  | |  |  | |
|  |  |  |  |  |  |  |  |  |  |  |
|  |  |  |  |  |  |  |  |  |  |  |
|  |  |  |  |  |  |  |  |  |  |  |
|  |  |  |  |  |  |  |  |  |  |  |
|  |  |  |  |  |  |  |  |  |  |  |

|  |  | | | | | | | | | |
| --- | --- | --- | --- | --- | --- | --- | --- | --- | --- | --- |
|  | |  | |  | |  | |  | |
|  | |  | |  | |  | |  | |
|  |  |  |  |  |  |  |  |  |  |
|  |  |  |  |  |  |  |  |  |  |  |
|  |  |  |  |  |  |  |  |  |  |  |
|  |  |  |  |  |  |  |  |  |  |  |
|  |  |  |  |  |  |  |  |  |  |  |
|  |  |  |  |  |  |  |  |  |  |  |
|  |  |  |  |  |  |  |  |  |  |  |

**S1 Table** Sensitivity analysis of the association of major congenital anomalies with antiepileptic drugs in the 1^st^ trimester of pregnancy after excluding women prescribed antiepileptic drugs for conditions other than epilepsy (N=258,321)

|  | **AEDs in the 1^st^ trimester** | | | | |
| --- | --- | --- | --- | --- | --- |
|  | **n exposed =1,024** | | | | |
|  | **n** | ***n/10,000*** | **aOR** | **95%CI** |  |
| **Any major anomaly** | 52 | 508 | 1.94 | 1.34-2.79 |  |
| Heart | 22 | 215 | 2.73 | 1.56-4.76 |  |
| Limb | 10 | 98 | 2.01 | 0.89-4.54 |  |
| Genital system | 8 | 78 | 2.02 | 0.81-5.04 |  |
| Nervous system | 4 | 39 | - | - |  |

Empty cells indicate there were fewer than five exposed cases, for which statistical analyses were not performed; aOR=odds ratio adjusted for maternal age, year of childbirth, maternal body mass index, smoking and socioeconomic status;

AEDs=antiepileptic drugs; 95%CI=95% confidence interval

and risk stratification according to whether high dose (at least 5mg daily) folic acid was prescribed*

|  |  | | | | | | | | | |
| --- | --- | --- | --- | --- | --- | --- | --- | --- | --- | --- |
|  | |  | |  | |  | |  | |
|  |  |  |  |  |  |  |  |  |  |
|  |  | |  | |  | |  | |  | |
|  |  |  |  |  |  |  |  |  |  |  |
|  |  |  |  |  |  |  |  |  |  |  |
|  |  |  |  |  |  |  |  |  |  |  |
|  |  |  |  |  |  |  |  |  |  |  |
|  |  |  |  |  |  |  |  |  |  |  |
|  |  |  |  |  |  |  |  |  |  |  |
|  |  | |  | |  | |  | |  | |
|  |  |  |  |  |  |  |  |  |  |  |
|  |  |  |  |  |  |  |  |  |  |  |
|  |  |  |  |  |  |  |  |  |  |  |
|  |  |  |  |  |  |  |  |  |  |  |
|  |  |  |  |  |  |  |  |  |  |  |
|  |  | |  | |  | |  | |  | |
|  |  |  |  |  |  |  |  |  |  |  |
|  |  |  |  |  |  |  |  |  |  |  |
|  |  |  |  |  |  |  |  |  |  |  |
|  |  |  |  |  |  |  |  |  |  |  |
|  |  |  |  |  |  |  |  |  |  |  |

trimester of pregnancy*

|  |  | | | |  |  | | | |  |  | | | |
| --- | --- | --- | --- | --- | --- | --- | --- | --- | --- | --- | --- | --- | --- | --- |
|  | | | |  |  | | | |  |  | | | |
|  |  |  |  |  |  |  |  |  |  |  |  |  |  |
|  |  |  |  |  |  |  |  |  |  |  |  |  |  |  |
|  |  |  |  |  |  |  |  |  |  |  |  |  |  |  |
|  |  |  |  |  |  |  |  |  |  |  |  |  |  |  |
|  |  |  |  |  |  |  |  |  |  |  |  |  |  |  |
|  |  |  |  |  |  |  |  |  |  |  |  |  |  |  |
